# Performance evaluation of the AiDx multi-diagnostic automated microscope for the detection of schistosomiasis in Abuja, Nigeria

**DOI:** 10.1038/s41598-023-42049-6

**Published:** 2023-09-08

**Authors:** Louise Makau-Barasa, Liya Assefa, Moses Aderogba, David Bell, Jacob Solomon, Rita Omohode Urude, Obiageli J. Nebe, Juliana A-Enegela, James G. Damen, Samuel Popoola, Jan-Carel Diehl, Gleb Vdovine, Temitope Agbana

**Affiliations:** 1 The Ending Neglected Diseases (END) Fund, New York, USA; 2 Unaffiliated, Lake Jackson, USA; 3 NTD Division, Federal Ministry of Health, Abuja, Nigeria; 4 CBM International, Cambridge, UK; 5 Medical Lab Department, University of Jos, Jos, Nigeria; 6 AiDx Medical Bv, Pijnacker, The Netherlands; 7 Delft University of Technology, Delft, The Netherlands

**Keywords:** Population screening, Infectious diseases, Parasitic infection, Urinalysis

## Abstract

In this research, we report on the performance of automated optical digital detection and quantification of *Schistosoma haematobium* provided by AiDx NTDx multi-diagnostic Assist microscope. Our study was community-based, and a convenient sampling method was used in 17 communities in Abuja Nigeria, based on the disease prevalence information extracted from the baseline database on schistosomiasis, NTD Division, of the Federal Ministry of Health. At baseline, samples from 869 participants were evaluated of which 358 (34.1%) tested *S. haematobium* positive by the reference diagnostic standard. Registered images from the fully automated (autofocusing, scanning, image registration and processing, AI image analysis and automatic parasite count) AiDx assist microscope were analyzed. The Semi automated (autofocusing, scanning, image registration & processing and manual parasite count) and the fully automated AiDx Assist showed comparable sensitivities and specificities of [90.3%, 98%] and [89%, 99%] respectively. Overall, estimated egg counts of the semi-automated & fully automated AiDx Assist correlated significantly with the egg counts of conventional microscopy (*r* = 0.93*, p* ≤ 0.001 and *r* = 0.89, *p* ≤ 0.001 respectively). The AiDx Assist device performance is consistent with requirement of the World Health Organization diagnostic target product profile for monitoring, evaluation, and surveillance of Schistosomiasis elimination Programs.

## Introduction

Schistosomiasis is a chronic parasitic disease that is caused by trematode blood flukes of the genus Schistosoma, with water snails serving as the intermediate host. Transmission has been reported from 78 countries, the majority of which are classified as low- or middle-income countries, according to (WHO) report and more than 700 million people are at risk of infection^1^. There are 2 major forms of schistosomiasis—intestinal (due to *Schistosoma mansoni and S. japonicum*) and urogenital (predominantly due to *S. haematobium*)*.* Common signs and symptoms of urogenital *Schistosoma haematobium* include a swollen belly, blood in the urine, stunted growth, cognitive impairment in children and infertility among adults of childbearing age. Advanced disease may sometimes present with fibrosis of the bladder and ureter, kidney damage and bladder cancer. Diagnosis is normally done through the detection of parasite eggs in urine or stool specimens using microscopy^2,3^.

Manual microscopy is the World Health Organization reference diagnostic method for the diagnosis of *S. haematobium* infection. Parasite-derived products (including eggs) in stool and urine can be identified and the eggs quantified. The egg count indicates the intensity of infection in a patient and can also reflect exposure, or prevalence in the target population. It is therefore important for therapeutic monitoring and surveillance^3–5^. However, manual microscopy is laborious, expertise-dependent and requires basic laboratory and power infrastructure, limiting its point-of-care application in endemic rural areas. Furthermore, the ratio of trained personnel to workload in endemic settings is low and this often results in a high workload per technician^6,7^. Innovative approaches to diagnosis that are easy-to-use and suitable for endemic resource-limited settings are required to diagnose infections and complement control and elimination efforts. Digital optical diagnostic devices with integrated Artificial Intelligence (AI) have been developed for the detection of *S. haematobium* eggs, soil transmitted helminths and other Neglected Tropical Diseases^8–17^. However, recent review of optical diagnostic devices reports that none of the developed devices had attained a technological readiness level required for use at point of care^9^.

Antigen-based diagnostic alternatives to conventional microscopy developed for schistosomiasis diagnosis include Point-Of-Care tests detecting Circulating Cathodic Antigens (POC-CCA) and the Up-Converting Particle Lateral Flow assay developed to detect Circulating Anodic Antigens (UCP-LF CAA) in either serum or urine^18,19^. These diagnostic techniques however require a level of infrastructure and training higher than microscopy and are non-specific in settings with *S. haematobium* only and on pregnant women. They also require standardization as a result of batch-to-batch variation. The use of Nucleic Acid Amplification Tests (NAATs) has been confined mainly to research settings as it is expensive and impractical at point of care^20–22^. Therefore, conventional microscopy remains the most widely used method.

In this work we assessed the performance of the novel multi-diagnostic AiDx Assist automated microscope for the quick detection of *Schistosoma haematobium*. The AI integrated innovative device was evaluated using urine samples to detect *S. haematobium* eggs in urine.

The Ethical Approval was received from the Federal Capital Territory (FCT, Nigeria) Health Research Ethics Committee (FCT, HREC) under approval number: FHREC/2022/01/102/05-07-22 and all research was performed in accordance with the relevant guidelines and regulations. The project was introduced in detail to the NTD Unit, Public Health Department, Federal Capital Territory Abuja (FCTA) who subsequently communicated details of the project to the local NTD officer in the selected area councils.

### Description of the AiDx assist microscope

**Figure 1:**
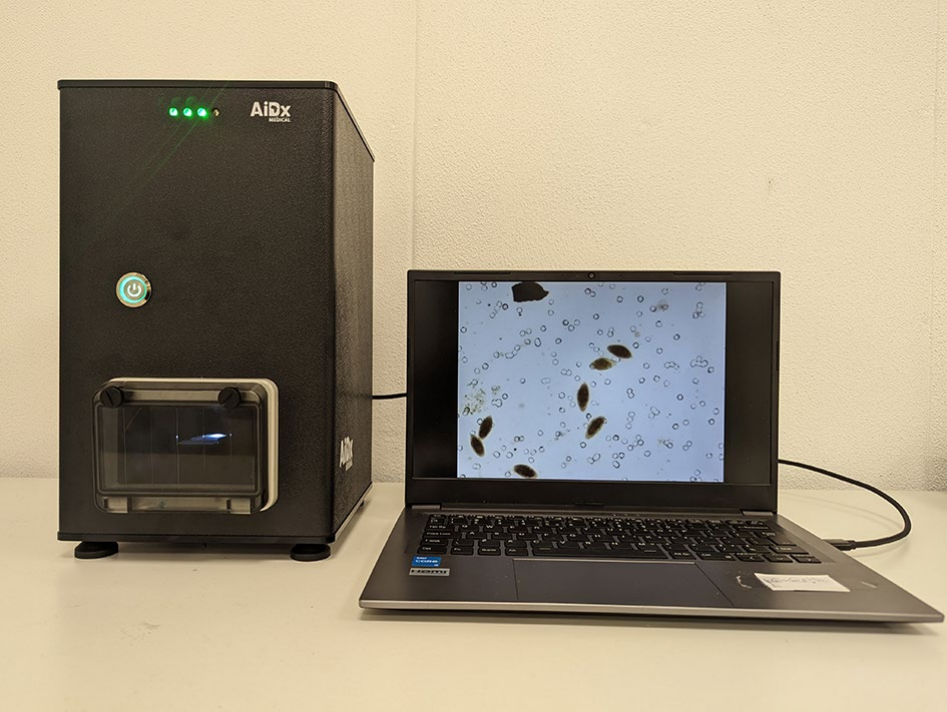
AiDx Assist fully automated microscopes designed for laboratory and field use.

The multi-diagnostic AiDx Assist Microscope device shown in Fig. 1, is a low-cost and compact automated single-slide scanner and microscope. The optical train consists of a 4× microscope objective (numerical aperture NA of 0.10 and a working distance of 18.0 mm). The current system model is designed with a Sony IMX 178 CMOS sensor 6.41 Mpix (3088 × 2076 pixel) with a pixel size of 2.40 μm.

The computer vision pipeline in this version of the device is powered by the U-Net segmentation model with a ResNet-18 network architecture. The approximate schistosome ova count was estimated using the predicted segmentation mask^23,24^. More recent approaches are being explored to improve the performance of the integrated algorithm.

The AiDx Assist multi-diagnostic device can detect microfilaria, S. *haematobium* and *S. mansoni eggs* in blood, urine, and stool samples respectively. Furthermore, the device has shown promising potential in the detection of malaria parasites and tuberculosis in prepared blood and sputum samples accordingly.

The AiDx Assist multi-diagnostic device can detect microfilaria, S. *haematobium* and *S. mansoni eggs* in blood, urine, and stool samples respectively.

The AiDx Assist Microscope can function in two modes at the choice of the operator:

#### Semi-automated mode

In the *semi-automated* mode, the automatic parasite count algorithm is disabled. The device operates without the use of the integrated Artificial Intelligence software. Images registered and stored in the system are visually examined for the presence of the target parasite. Operating the device in this mode is very useful for experienced laboratory scientists who prefer to gain control of the device and navigate through the stored sample scan to detect the target of their interest without dependence on the AI. The integrated sample autofocusing mechanism and scanning system control algorithm reduces the time, error, fatigue, and other limitations associated with manual microscopy.

#### Fully automated mode

The *fully automated* AiDx Assist include an autofocusing algorithm which obtains an in-focus image obtained in a z-stack of image, a sample scanning algorithm to scan defined samples area. It also includes image registration, processing, AI image analysis and automatic parasite count. Detected targets are segmented and segmented images are stored in a folder for easy access and validation by the AiDx system operator. The AI Assist software outputs the number of estimated ova for the operator to validate and confirm the presence of the segmented Schistosoma ova.

## Results

Three detection methods were analyzed: (i) The conventional microscopy which is the reference standard (ii) The expert analysis of the result of Semi-automated AiDx Assist and (iii) The fully automated AiDx Assist digital microscope with automated image interpretation. The three methods independently identified 358 (41.2%), 324 (37.3%) and 315 (36.2%) of the total slides as positive for *S. haematobium* respectively as shown in Table 1. The egg count estimates per 10 mL of urine ranged from 1 to 2100 eggs/10 mL for conventional microscopy, 1 to 5451 eggs/10 mL for semi-automated, and 1 to 4064 eggs/10 mL for fully automated AiDx Assist. Compared with conventional microscopy, semi-automated and the fully automated AiDx assist showed an overall accuracy of 94% and 95%, respectively (Table 1).

**Table 1:** *S. haematobium* egg count by conventional manual microscopy and automated AiDx assist microscope.

Conventional microscopy (reference)		Positive (n = 358)	Negative (n = 511)	Total (n = 869)
Semi-automated AiDx assist	Positive	324	10	334
	Negative	34	501	535
Fully automated AiDx assist	Positive	315	6	321
	Negative	43	505	548

The sensitivities of the semi-automated and fully automated AiDx Assist for the detection of *S. haematobium* eggs were comparable; 90.5% and 88%, respectively. Details of evaluated performance metrics is shown in (Table 2). Further review of the data showed that there is no erroneous choices overlap between the semi-automated and the fully-automated AiDx Assist. For instance, there was zero overlap between the false positive and the false negative samples detected by the semi and fully automated AiDx Assist respectively.

**Table 2:** Evaluated performance metric result for the AiDx assist microscope.

Conventional microscopy	Semi-automated AiDx assist (334—positive cases)				Fully automated AiDx assist (321—positive cases)			
	Sen (%)	Spec (%)	PPV (%)	NPV (%)	Sen (%)	Spec (%)	PPV (%)	NPV (%)
AiDx assist microscope								
All samples with *S. haematobium* infection (N = 358)	90.5	98	99	93	88	99	98	92
Low and high intensity detection by semi/fully-automated AiDx NTDx								
Low intensity infection* (N = 244)	86	–	–	–	82.4	–	–	–
High-intensity infection (N = 114)	100	–	–	–	100	–	–	–

*Sen* sensitivity, *Spec* specificity, *NPV* negative predictive value, *PPV* positive predictive value.

*< 50 eggs/10mL urine.

To characterize the performance of the AiDx Assist Microscope in the detection of low-intensity and high intensity infection, we compare the semi-automated and the fully automated functionalities of the AiDx Assist microscope with reference conventional microscopy. In this case, the conventional microscopy classified 244 (68.1%) as low-intensity infection and (31.8%) as high-intensity infection. The fully automated and Semi-automated AiDx Assist classified 246 (76.6%) and 237 (70.9%) as low-intensity infection respectively, 75 (33.9%) and 97 (29%) were classified as high-intensity infection. Sensitivities of the fully automated and semi-automated AiDx Assist for low-intensity infections were 86% and 82.38%, which increased to 100% for high-intensity infections in both cases accordingly.

**Figure 2:**
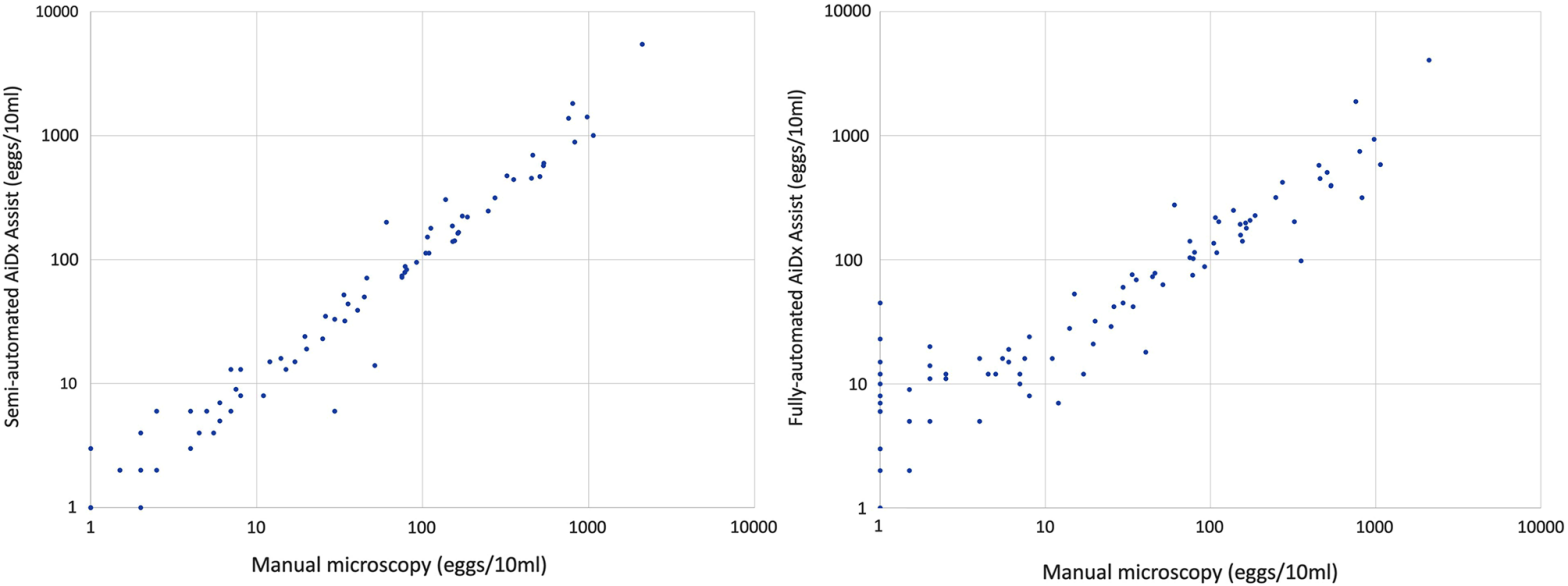
Graphic representation of the correlation between manual microscopy (reference) and AiDx assist methods. Data shown for (**a**) semi automated AiDx assist versus manual microscopy (reference) and (**b**) fully-automated AiDx Assist versus manual microscopy (reference).

In Fig. 2 the graphical representation of the correlation between fully/semi-automated AiDx Assist Microscope and the reference manual microscopy method is shown. The correlation between the fully automated AiDx Assist and the reference manual microscopy was lower (Pearson 0.89, p < 0.001) compared to the correlation between the Semi-automated AiDx Assist and reference manual microscopy (Pearson 0.93, p < 0.001). No significant change was observed in the correlation coefficient estimates when the outliers were removed. This implies that the model is robust to the removal of the outliers.

**Figure 3:**
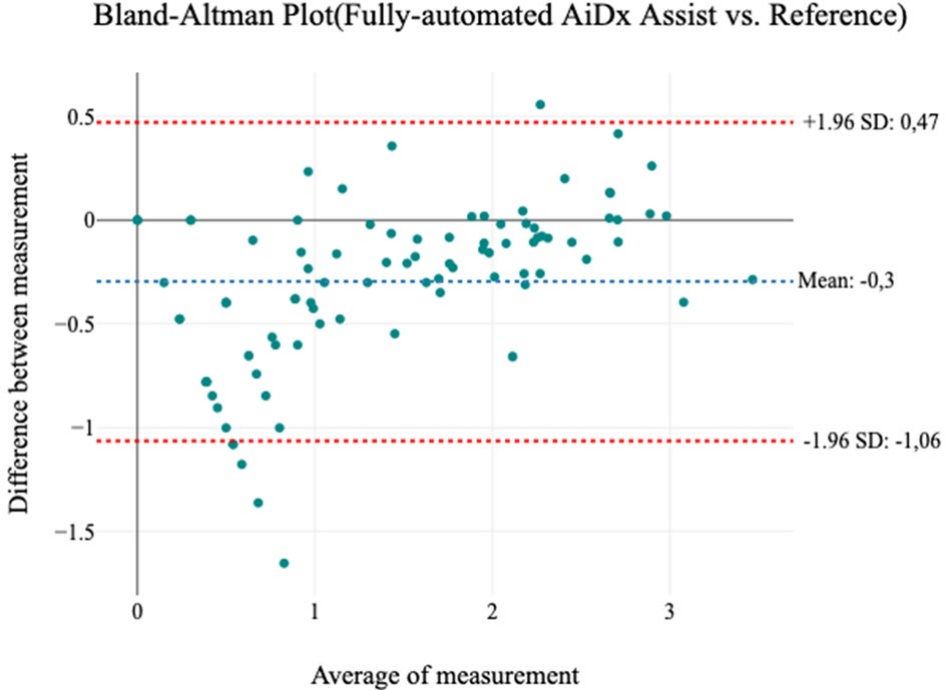
Bland–Altman plots showing the level of agreement between the manual microscope and semi-automated AiDx Assist. The plot shows strong agreement in measurement with (r = 0.93, p ≤ 0.001).

**Figure 4:**
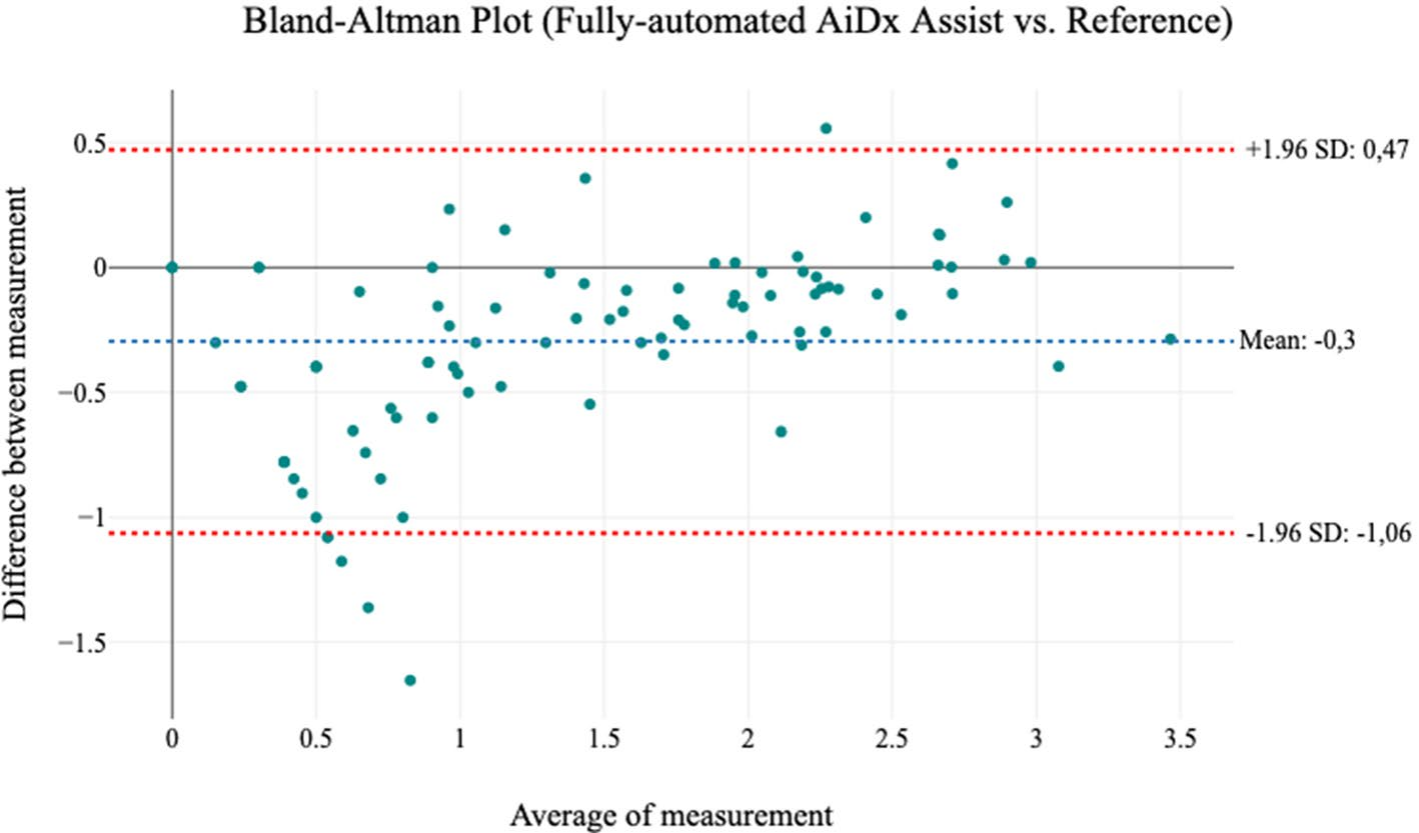
Bland–Altman plots showing the level of agreement between the manual microscope and fully automated AiDx Assist Microscope. The plot shows strong agreement in measurement with (r = 0.89, p ≤ 0.001).

To further validate the agreement between the AiDx Assist and the reference manual microscopy, Bland–Altman analysis was performed on 93 samples collected from a highly endemic community. The sample size was chosen to represent the range of infection from low, medium, and high infection accordingly. Graphical representation of the correlation between manual microscopy (reference) and fully/semi-automated AiDx Assist parasitemia count data is shown in Figs. 3 and 4. A logarithmic scale (log(x + 1)) transformation of the data is used to avoid the log(x) approaching negative infinity as x approaches zero.

The Bland–Altman plot shows a strong agreement. Removing the outliers in the plot did not correspond to any significant change in the computed correlation coefficient.

### False positives—misclassified samples

With respect to the reference standard conventional microscopy, a third trained reader examined digital images of all (869) samples registered by the semi-automated AiDx Assist microscope (the 3rd microscopist was blinded to the results obtained from the reference microscopy and the fully automated AiDx Assist digital microscope to avoid any form of bias). The result was compared to the average reading of the expert microscopist. 10 out of the 10 false positives were confirmed to be true positive samples. The specific images were sent to the expert microscopist who validated the schistosome ova in the registered images. We observe however that the samples had very low egg count range (1–14 eggs/10 mL of urine).

## Discussion

This study focused on the field performance evaluation of the AiDx Assist for the detection of *S. haematobium* eggs in infected urine samples. The system has been evaluated as (i) A semi-automated AiDx Assist microscope and (ii) fully automated AiDx Assist microscope. The integration of the AI algorithm as described in the previous section increased the specificity of the fully automated AiDx Assist. This is a unique hybrid (AI + Human operator) feature that demonstrates increase in specificity. Complete dependence on the AI analysis could result in low specificity which could significantly compromise the quality of the result.

The semi-automated AiDx Assist microscope demonstrated better performance than the manual microscope as it detected additional positive cases which were missed by the expert manual microscopy. Underestimation of the intensity of infection was observed in some cases. This could be due to two primary reasons: Firstly, the autofocus technique implemented fails to converge to the optimal in-focus plane consistently. This increases the AI algorithm’s poor generalization in defocused images. Secondly misrecognition of overlapping eggs as a single egg by the AI algorithm. Complete sample analysis was achieved in 15 min. During this field study performance evaluation this was a limitation of the AiDx Assist. In the current system development iteration of the device however, complete sample analysis is achieved in less than 6 min. While the AiDx Assist provides clear digital images of the stool parasite, a functional AI algorithm to detect *S. mansoni* and STHs is currently being developed.

The semi-automated AiDx Assist and fully automated AiDx Assist has proven to hold great potential for future and timely diagnosis of schistosomiasis as it has good sensitivity, specificity, positive and negative predicted values respectively. The AiDx Assist device performance evaluated in this study is consistent with requirement of the World Health Organization Diagnostic Target Product Profile for monitoring, evaluation, and surveillance of Schistosomiasis. The usefulness of this novel AI-based AiDx Assist device will be of great value in schistosomiasis diagnosis, evaluation, surveillance and may be incorporated into elimination programs agenda.

## Methods

### Study design and population

Participants aged 5 and above were eligible to participate in this study. Study was community-based, and a convenient sampling method was used in 17 communities: 9 communities were selected from Gwagwalada Area council and 8 from Abuja Municipal Area Council (AMAC) of the Federal Capital Territory, Abuja Nigeria. The Area councils were selected based on the disease prevalence information extracted from the baseline database on schistosomiasis, NTD Division, of the Federal Ministry of Health. Participants collected their urine samples in the bathroom at collection sites. These were stored in temperature-controlled storage boxes (2–6 °C) and transported to the laboratory immediately after collection. The sample size of the study population was computed using the Cochran formula^24^. Given the baseline prevalence for Gwagwalada and AMAC at 51.9% and 21.12% respectively, a total of 869 (509 and 360) respondents were sampled from Gwagwalada and AMAC. They were randomly selected from 10 and 7 Communities in Gwagwalada and AMAC respectively. Smaller samples were sampled in the Area Council with lower prevalence to ensure that enough positive samples are collected. The power of the diagnostic test required to achieve adequate sensitivity or specificity is calculated based on the formula outlined in Jones et al.^25^.

### Sample collection process

**Figure 5:**
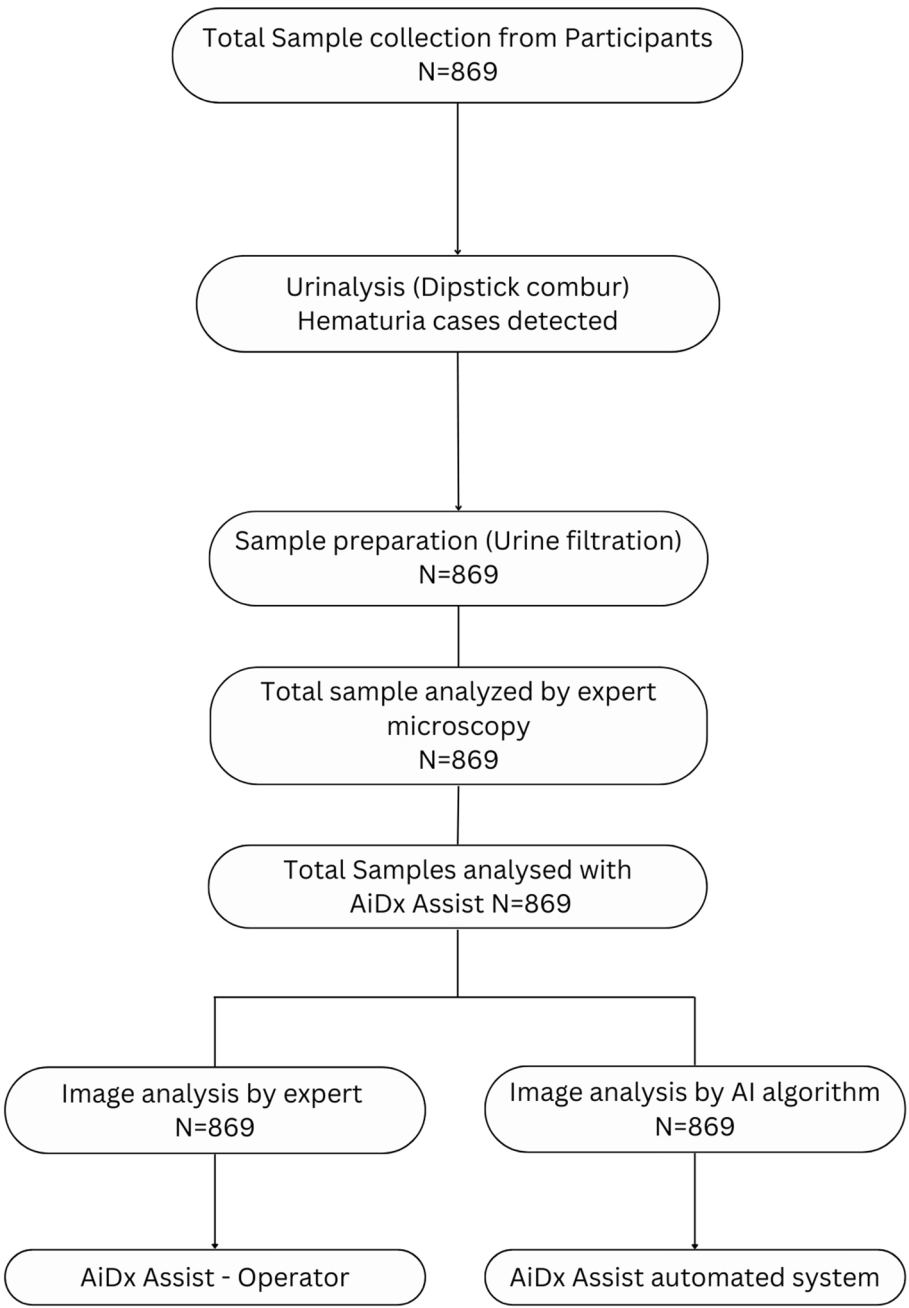
Flow chart of urine sample collection and analysis comparing conventional microscopy with the AiDx assist automated and digital result analysis by expert.

Community consent forms which were filled and signed by community leaders on behalf of community members according to standard government protocol were administered by the data manager of the NTD unit, the Public Health Department of the Federal Capital Territory Abuja (FCTA). The hardcopy version of all the consent forms were then transferred to an electronic format and uploaded to the cloud server platform. To ensure full confidentiality, each participant was assigned a specific unique identification number. Each selected participant was provided with a capped sterile specimen bottle and instructed to fill it with about 20 mL of clean catch, midstream urine. Participants were subjected to a little exercise to agitate their bladder and carefully instructed with illustration aid. The bottles were labeled with unique identification (ID) numbers and samples were collected between 10.00 am and 14.00 h. The age and sex of each participant were noted and recorded. Samples were transported to the laboratory and processed 1–2 h after collection. In the laboratory, each urine sample was tested for hematuria using urinalysis reagent strip (Combi 9 Dipstick), then homogenized by gentle agitation and was filtered using urine filtration technique to concentrate eggs of schistosome on a membrane filter as described by WHO, 2010. The number of eggs were counted by a microscopist and recounted by a second independent microscopist both of whom were blinded to the urinalysis results to reduce margin of error and results expressed as number of eggs per 10 mL of urine. Same urine slide was then passed to the AiDx machine for image-based microscopy and count. The data collection form which contained data entry space for urine results from Dipstick and microscopists were submitted daily at the AiDx Medical Laboratory office after sample analysis by the technicians and the laboratory scientist. Quality control was instituted, and laboratory results verified by senior laboratory scientists and Federal Ministry of Health (FMoH) supervisors to ensure consistency in sample preparation and examination. A comparative flow chart of the sample collection and analysis of the urine samples is shown in Fig. 5.

### Detection of *Schistosoma haematobium* eggs by microscopy and the AiDx assist

**Figure 6:**
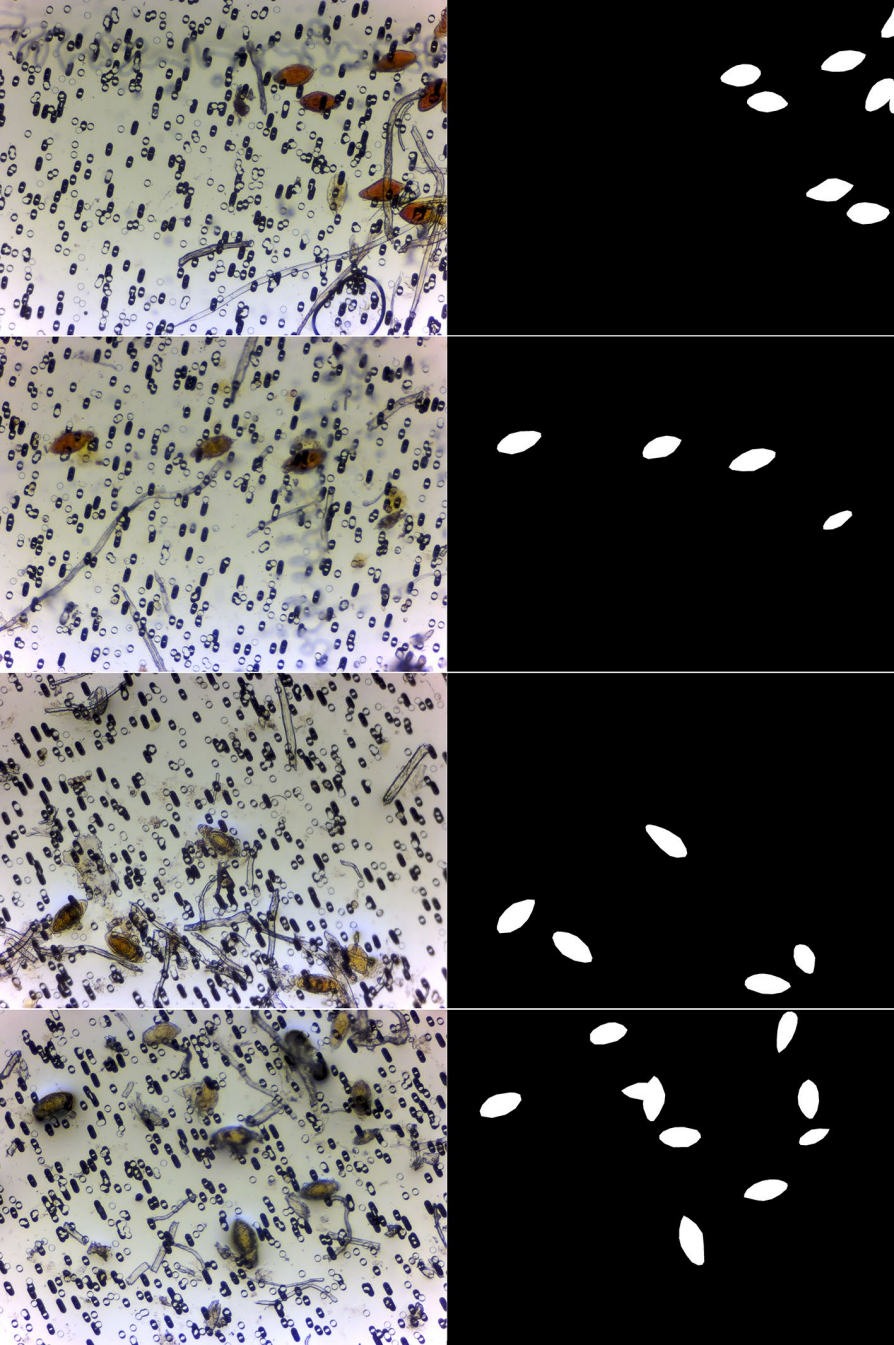
Registered images of *S. haematobium* eggs. The approximate ova count is estimated using the predicted segmentation mask. Images on the left are the captured images and images on the right are the annotations.

Prepared samples were read blinded by two expert laboratory scientists from the Federal Ministry of Health Schistosomiasis Program using a 10/40(×) objective on a Leica microsystems DM 300 microscope. The same urine slide was then passed to the automated AiDx Assist for image-based microscopy and count. Example images registered by the fully automated AiDx Assist across the range of mean egg counts for both methods is shown in figure below (Fig. 6). The approximate ova count is estimated using the predicted segmentation mask. Images on the left are the captured images and images on the right are the annotations.

**Figure 7:**
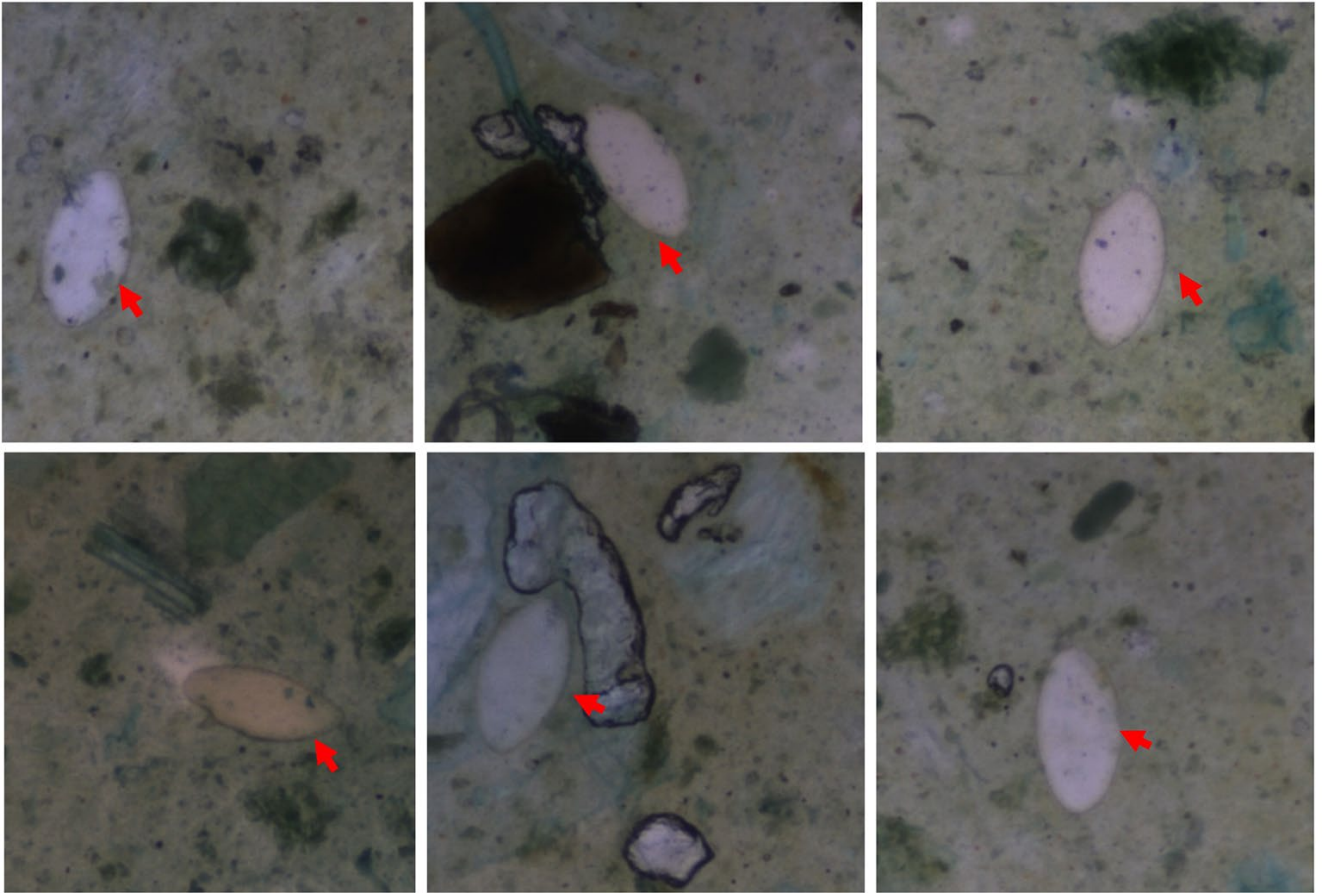
Registered images of S. mansoni eggs in stool samples. The characteristic terminal spine is located at the side of the ova. Although the stool samples contain lots of debris and potential artifacts, the AiDx automated microscope sufficiently resolves the desired target.

Figure 7 shows *S. mansoni* eggs detected in stool samples by the automated AiDx Assist. The fecal samples are prepared based on the Kato Katz technique. AI algorithm for the detection of the *S. mansoni* in registered images has been developed for testing in a planned upcoming field study.

### Statistical analysis

Qualitative agreement between the automated AiDx Assist and conventional microscopy was assessed. Egg counts were categorized as low-intensity infection (< 50 eggs/10 mL of urine) or high-intensity infection (> 50 eggs/10 mL urine). Data transformation was performed to increase the data cohesion. The linear association in terms of egg counts (eggs/10 mL) between the different optical procedures was estimated using Pearson’s correlation coefficient (r). Bland–Altman analysis was performed for quantitative assessment of the agreement between manual microscopy (reference) and fully/semi-automated AiDx Assist using the Datatab statistical calculator.

### Ethics statement

The study involving human participants was reviewed and approved by the Federal Capital Territory (FCT, Nigeria), Health Research Ethics Committee (FCT, HREC). Written Informed consent to participate in this study was provided by the participants.

## Data Availability

The datasets generated and analyzed for this study are not publicly available due to privacy reason but are available from the corresponding author upon reasonable request.
